# Oral Administration of Cannabis and Δ-9-tetrahydrocannabinol (THC) Preparations: A Systematic Review

**DOI:** 10.3390/medicina56060309

**Published:** 2020-06-23

**Authors:** Lourdes Poyatos, Ana Pilar Pérez-Acevedo, Esther Papaseit, Clara Pérez-Mañá, Soraya Martin, Olga Hladun, Adrià Siles, Marta Torrens, Francesco Paolo Busardo, Magí Farré

**Affiliations:** 1 Clinical Pharmacology Department, Hospital Universitari Germans Trias i Pujol and Institut de Recerca Germans Trias (HUGTiP-IGTP), 08916 Badalona, Spain; lpoyatos@igtp.cat (L.P.); epapaseit.germanstrias@gencat.cat (E.P.); cperezm.mn.ics@gencat.cat (C.P.-M.); smartins.mn.ics@gencat.cat (S.M.); ohladun.germanstrias@gencat.cat (O.H.); mfarre.germanstrias@gencat.cat (M.F.); 2 Departments of Pharmacology, Therapeutics and Toxicology and Department of Psychiatry, Universitat Autònoma de Barcelona, 08193 Cerdanyola del Vallés, Spain; mtorrens@parcdesalutmar.cat; 3 Pharmacy Department, Hospital Universitari Germans Trias i Pujol and Institut de Recerca Germans Trias (HUGTiP-IGTP), 08916 Badalona, Spain; asiles@igtp.cat; 4 Drug Addiction Program, Institut de Neuropsiquiatria, Parc de Salut Mar and Institut Hospital del Mar de Recerca Mèdica (PSMAR-IMIM), 08003 Barcelona, Spain; 5 Department of Excellence-Biomedical Sciences and Public Health, Università Politecnica delle Marche, 60121 Ancona, Italy; f.p.busardo@staff.univpm.it

**Keywords:** oral cannabis, oral THC, medical use, cannabis edibles, pharmacokinetics

## Abstract

*Background and objective*: Changes in cannabis legalization regimes in several countries have influenced the diversification of cannabis use. There is an ever-increasing number of cannabis forms available, which are gaining popularity for both recreational and therapeutic use. From a therapeutic perspective, oral cannabis containing Δ-9-tetrahydrocannabinol (THC) and cannabidiol (CBD) is a promising route of administration but there is still little information about its pharmacokinetics (PK) effects in humans. The purpose of this systematic review is to provide a general overview of the available PK data on cannabis and THC after oral administration. *Materials and Methods*: A search of the published literature was conducted using the PubMed database to collect available articles describing the PK data of THC after oral administration in humans. *Results*: The literature search yielded 363 results, 26 of which met our inclusion criteria. The PK of oral THC has been studied using capsules (including oil content), tablets, baked goods (brownies and cookies), and oil and tea (decoctions). Capsules and tablets, which mainly correspond to pharmaceutical forms, were found to be the oral formulations most commonly studied. Overall, the results reflect the high variability in the THC absorption of oral formulations, with delayed peak plasma concentrations compared to other routes of administration. *Conclusions*: Oral THC has a highly variable PK profile that differs between formulations, with seemingly higher variability in baked goods and oil forms. Overall, there is limited information available in this field. Therefore, further investigations are required to unravel the unpredictability of oral THC administration to increase the effectiveness and safety of oral formulations in medicinal use.

## 1. Introduction

### 1.1. Cannabinoids

Cannabis is the most widely used illicit drug worldwide, only surpassed by alcohol and tobacco when also considering legal substances. Recent investigations have highlighted the therapeutic potential of cannabis, resulting in a resurgence of its consumption for medical purposes. Although cannabis continues to be used mostly for recreational purposes, people increasingly consume it to benefit from its therapeutic properties [1,2,3].

Δ-9-Tetrahydrocannabinol (THC) is the principal source of the psychoactive effects associated with cannabis use [3]. These effects result from the activity of THC as a partial agonist of the cannabinoid receptor CB1, which is primarily located in the central nervous system, and CB2, which is predominantly expressed in the peripheral tissues [4]. THC has observable effects on behavior, nociception, and appetite, as well as anti-inflammatory, antitumor, and antiemetic properties. THC is also responsible for the psychotropic effects and addictive and reinforcing properties of cannabis [5].

The other predominant component in the cannabis plant is cannabidiol (CBD), which is the primary cannabinoid in fiber-type hemps. Unlike THC, CBD does not have a direct effect on the receptors (CB1 and CB2) responsible for cannabis’ psychoactive effects [6,7,8], although recent evidence has demonstrated the negative allosteric activity of CBD on CB1 [9].

CBD is believed to attenuate THC’s psychotropic effects, thereby enhancing the safety (or safety profile) of cannabis products containing both cannabinoids. However, this interaction is not fully understood. Previous studies found that CBD does not alter THC’s subjective effects [10,11,12], but a recent study reported an increase in plasma THC concentration and a slight exacerbation of THC-induced impairment in the presence of CBD [13].

Other cannabinoids may play a role in the overall effects of cannabis, such as Δ-8-tetrahydrocannabinol, cannabinol (CBN), cannabicyclol (CBL), cannabichromene (CBC), and cannabigerol (CBG). However, these cannabinoids have fewer psychotropic effects than THC [14].

### 1.2. Therapeutic Uses of Cannabis

Cannabinoids exert most of their biological effects through interactions with the endocannabinoid system. Their wide range of effects makes cannabinoids good candidates for treating many ailments, including nausea, loss of appetite, neuropathic pain spasticity, epilepsy, chronic pain, etc. [5]. However, cannabinoids are currently typically prescribed as adjuvant treatments or after a patient does not respond well to first-line treatments.

The number of countries that have legalized therapeutic cannabis use (medical cannabis) has grown in recent years. In the Netherlands, the drastic increase in the prevalence of medical cannabis prescriptions can be explained by the emergence of new formulations, especially cannabis oils [15], which have become the preferred option for therapeutic use [16].

When considering the purpose of consumption, for medical cannabis users, edible cannabis is one of the most common consumption modes, along with vaporization [17,18,19], contrary to recreational users, who are more likely to smoke or vaporize [18].

### 1.3. Oral Cannabis and THC and other Routes of Administration

Recreational cannabis is mainly consumed by smoking, which involves combusting the herbal cannabis present in a joint, blunt, pipe, bubbler, or bong/water pipe, among other forms. The psychoactive effects of THC appear in less than a minute after consumption. From the limited evidence available, it appears that smoking cannabis may be associated with respiratory diseases, as smoked cannabis contains several toxins and carcinogens also found in tobacco smoke [20]. No country that has authorized the medical use of cannabis recommends smoking as a method of consumption.

An alternative inhalation-based form that has become popular in recent years is using a vaporizer. This technique is considered less noxious than regular smoking, as vaporizing does not produce the pyrolytic compounds derived from combustion of the dried herb or extract, such as polycyclic aromatic hydrocarbons. However, vaporizers have been recently associated with acute respiratory illness, now referred to as e-cigarette or vaping product-use associated lung injury (EVALI) [21,22]. The cause of this condition is currently under investigation, although there is evidence implicating the vitamin E acetate used as a diluent in vaporizer liquids [23].

Changes in cannabis legalization in several countries have influenced the emergence of a variety of edible products containing cannabis, which have increasing popularity. At present, edible products are now available in new formats resembling sugary snacks (hard and soft candies) and baked goods (brownies, cookies), which appeal especially to young people. From a therapeutic perspective, oral cannabis intake is promising due to its long-lasting drug effects, easy administration, and reduced toxicity derived from pyrolytic by-products. To date, the limited information available describes a slow and erratic absorption, seemingly showing higher bioavailability in oil formulations [24].

### 1.4. Oral Cannabinoid Preparations

Medical cannabis refers to a broad range of products and preparations that contain cannabis and cannabinoids for therapeutic purposes. Several medical products with marketing authorization contain THC as their main component, including dronabinol (synthetic THC), commercialized as oral capsules (Marinol^®^) or as an oral solution (Syndros^®^), and nabilone (a synthetic THC analogue), which is marketed as oral capsules (Cesamet^®^ or Canemes^®^). Nabiximols (Sativex^®^), available as a buccal spray, also includes CBD in its formulation [14,24,25,26]. However, few cannabis products have sufficient evidence to obtain approval for therapeutic use in the USA and several European countries.

Formulations derived from the *Cannabis sativa* plant that do not have marketing authorization for medical use are known as “cannabis preparations”. This term encompasses raw cannabis, compound preparations, and standardized cannabis preparations, which include cannabis flowers, granulates, and oil extracts [27].

In the Netherlands, cannabis is produced in five standardized strains that are commercially available and classified by their THC and CBD content [28]. In Italy, the Ministry of Health authorized the commercialization of standardized cannabis (FM2, 5–8% THC and 7–12% CBD) for medical purposes, which is produced by the Military Pharmaceutical Institute in Florence [29]. As per recommendations, this medical cannabis is consumed in decoctions or oils following standardized indications [30]. In 2018, a new strain of medical cannabis began production (FM1, 13–20% THC and <1% CBD), but no therapeutic indications are currently authorized for this new strain [31,32]. In Canada, medical cannabis is manufactured under a public license, mostly in the form of oil. Cannimed^®^ can be purchased as oil capsules, oil, dried flowers, or in a topical form, allowing oral, vaporized, or topical administration [33,34].

Edible cannabis is associated with a high rate of emergency department visits, mainly due to gastrointestinal symptoms, intoxication, and psychiatric effects [35]. The high variability of oral THC absorption and the delayed onset of effects can lead to the overconsumption of edible preparations, especially among naive users.

Considering that cannabinoids are proposed to alleviate a wide range of ailments, the identification and interpretation of their pharmacokinetics (PK) are essential for their use as pharmaceutical products. The purpose of this review was to examine the available data on THC PK after the oral administration of cannabis and THC, as reported in humans.

## 2. Materials and Methods

This systematic review was performed according to PRISMA (Preferred Reporting Items for Systematic Reviews and Meta-Analyses) guidelines [36].

Potential studies were systematically searched and identified by one of the authors (L.P.). The study selection was jointly conducted by two of the authors (L.P. and M.F.). After reading the summary of each study, in case of a disagreement, the final decision was reached by consensus. The data were collected by the author L.P. and reviewed by the other authors (M.F. and A.P.P.).

A search of the published literature was conducted using the PubMed database up to March 2020. The keywords used for the search were “oral cannabis”, “edible cannabis”, “oral THC”, “pharmacokinetic”, “blood collection”, “dronabinol”, “synthetic THC”, “plasma levels”, and “Cmax”. To be included, studies had to follow a pharmacokinetic model, include at least the maximum plasma concentration values (Cmax) and time needed to reach the maximum concentrations (Tmax) as PK parameters, and examine oral administration, although they could also include other routes of administration. Single-dose studies were preferred, but multiple-dose studies were included if the blood was collected at various time points following initial dosing. Only articles whose abstracts met our selected criteria were selected. Animal studies, articles focusing on routes of administration other than oral, nabilone (a synthetic cannabinoid), nabiximols, and studies of CBD administration were excluded from the review. Studies featuring the administration of nabiximols were only included if their preparations were compared to those of other oral cannabis/THC preparations.

Only articles written in English were selected. All articles were reviewed independently by the authors to determine their relevance in the framework of the current study.

The following data were collected from the reviewed articles: Design of the study, number of participants, gender, previous experience with cannabis, route of administration, product containing THC, dose of THC, basic PK measurements (maximum concentration, or Cmax, and time to achieve maximum concentrations, or Tmax) in plasma or blood, and pharmacological effects (if measured).

In order to evaluate a relationship between the administered dose and peak concentrations (Cmax), Spearman correlations were conducted between doses of THC and Cmax values in each formulation group. A linear regression procedure that allows for the calculation of correlation coefficients was used. Analyses were performed using GraphPad Prism 5.

## 3. Results

**Figure 1:**
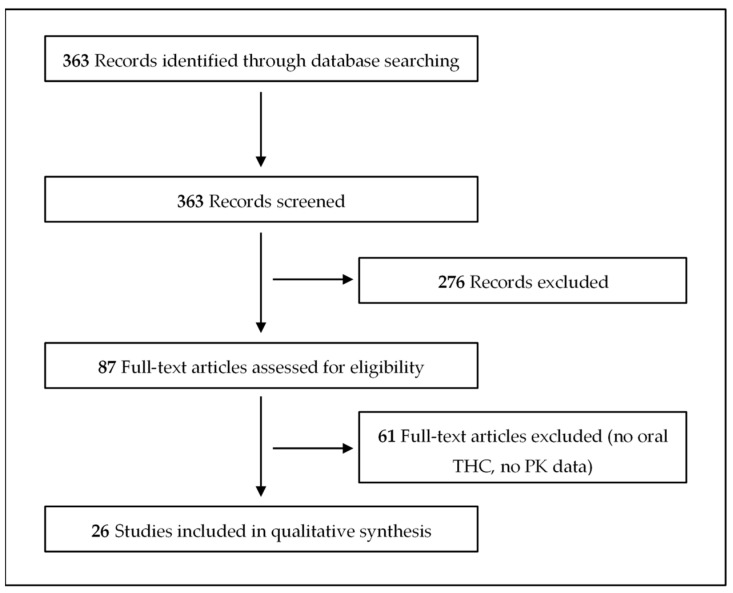
Flow chart for the study retrieval and selection.

The literature search yielded a total of 363 results, 26 of which met our inclusion criteria (Figure 1) [37,38,39,40,41,42,43,44,45,46,47,48,49,50,51,52,53,54,55,56,57,58,59,60,61,62]. These studies are summarized in Table 1, Table 2, Table 3 and Table 4. Besides the oral administration route, cannabis was taken in by other routes in eight of the studies, including sublingual, respiratory/inhaled, buccal, intravenous, and oropharyngeal routes. THC can be present in various dosage forms, each with different PK properties, which are crucial to know for a molecule intended for therapeutic applications. The oral absorption of THC was studied using oil capsules, tablets, baked goods (brownies and cookies), oils, and decoctions. However, there was no information on other products containing cannabis, including candies and chocolates.

Overall, these studies showed remarkable heterogeneity in their designs and the conditions under which they were conducted. Most studies included small sample sizes, and not all of them included subjects of both genders. There were also discrepancies in the cannabis experience levels of the participants and their health statuses, since patients with diverse pathologies (HIV, chronic pancreatitis, and medication overuse-related headaches) were targeted in several studies. Most of the studies involved THC administration alone, although THC was administered in combination with CBD in eight studies. Apart from other cannabinoids, THC was also administered combined with other drugs, such as megestrol acetate, naltrexone, and morphine.

Some studies included evaluations of physiological and/or subjective effects after administration of the different preparations. THC/cannabis was found to produce its prototypical effects, presenting mild changes in blood pressure or heart rate, increases in scores of high and positive effects, increased feelings of sedation/drowsiness, and mild impairment of psychomotor performance. See Table 1, Table 2, Table 3 and Table 4 for detailed descriptions of these effects in different studies according to various formulations.

### 3.1. Capsules

Cannabis capsules usually contain cannabis or synthetic THC (dronabinol) in oil due to its higher bioavailability (see also the oil section). In our search, 14 studies investigated cannabis/THC/dronabinol administration dissolved in oil capsules, thus representing the most frequently studied dosage form among all oral formulations [37,38,39,40,41,42,43,44,45,46,47,48,49,50].

The dose of THC contained in the oil capsules in single-dose studies ranged from 5 to 90 mg. The Cmax in plasma ranged from 0.42 to 29.9 ng/mL, and the Tmax ranged from 0.78 to 4 h. PK differences were examined after administering the same cannabinoid doses (10.8 mg of THC and 10.0 mg of CBD) using THC and CBD-piperine-pro-nanolipospheres (THC-CBD-PNL) capsules, which are an alternative to oil capsules, and an oromucosal spray (Sativex^®^). THC-CBD-PNL produced a three-fold increase in Cmax compared to Sativex^®^ (5.4 and 1.8 ng/mL of THC, respectively) and a faster absorption of cannabinoids (a Tmax of 1 h and 2 h for THC and of 1 h and 2 h for CBD, respectively) [51]. See Table 1 for the specific results.

**Figure 2:**
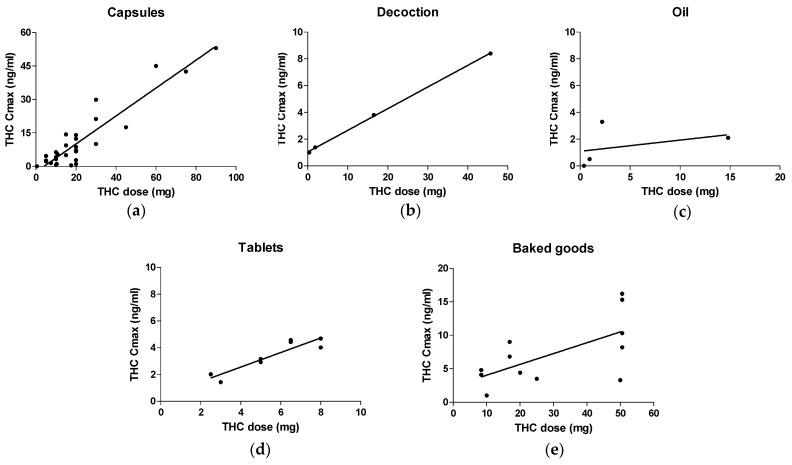
The correlation between the dose of administered THC and the maximum concentration (Cmax) of THC in plasma following administration of capsules (**a**), decoctions (**b**), oils (**c**), tablets (**d**), and baked goods (**e**).

The correlation between the administered dose and Cmax resulted in a Pearson’s *r* value of 0.9271 and a coefficient of determination (R^2^) of 0.8596 (Table 5, Figure 2). The THC Cmax increased proportionally by increasing the doses of THC.

### 3.2. Oil

In previous studies, oil extracts showed greater cannabinoid extraction efficiency than water [31]. In addition to a higher bioavailability, oil formulations are considered suitable solvents to compose a THC therapeutic preparation. In this section, we only considered the administration of oil-based cannabis (see above for capsules containing oil). Only three studies were found in which oil was directly ingested [43,52,53]. Among these three studies, only two reported single-dose administrations. In one of these studies, subjects received 2.2 mg of THC and 2.3 mg of Δ-9-tetrahydrocannabinolic acid A (THCA-A), obtaining a Cmax of 3.29 and 65.36 ng/mL, respectively, and a Tmax of 1.28 and 1.33 h in plasma [52]. The other study reported data on one healthy individual treated with a cannabis decoction and oil (pilot study). The subject received 0.45 mL of oil containing 0.95 mg of THC, 1.5 mg of THCA-A, 0.86 mg of CBD, and 2.8 mg of cannabidiolic acid (CBDA). The THC and THCA-A Cmax in serum following oil administration were 0.5 and 40.3 ng/mL, respectively, with a Tmax of 2.0 h for both cannabinoids [53]. See Table 2 for the specific results.

In this formulation, among the three studies included, the administered dose of THC showed a weak and not significant correlation with the Cmax (Pearson’s *r* = 0.3806, *p* value = 0.6194) (Table 5, Figure 2). 

### 3.3. Decoctions

Decoctions are also called “tea” in several articles. Only three studies examining cannabinoid PK after cannabis decoction administration were retrieved, including one study with a milk decoction [41,52,53]. For the milk decoction, two doses were selected—a low THC dose of 16.5 mg and a high dose of 45.7 mg, achieving a Cmax of 3.8 and 8.4 ng/mL, respectively, and a Tmax of 1 h in plasma for both doses [41]. In another study, cannabis was boiled in water, obtaining a decoction composed of 1.85 mg THC and 2.22 mg of THCA-A. After consumption of this decoction, the THC reached a mean Cmax of 1.38 ng/mL with a Tmax of 1.28 h, whereas THCA-A reached a mean Cmax of 48.92 ng/mL in plasma with a Tmax of 1.22 h [52]. In the pilot study mentioned in the Oil section, the subject received 100 mL of a cannabis decoction containing 0.36 mg of THC, 1.6 mg of THCA-A, 0.42 mg of CBD, and 4 mg of CBDA. This oral dose resulted in a Cmax in the serum of 1.0 ng/mL of THC and 72.4 ng/mL of THCA-A, with a Tmax of 2.0 h [53]. See Table 2 for the specific results.

Despite the few studies found, cannabis decoctions showed a significant strong correlation between the dose of THC and peak plasma, with a Pearson’s *r* of 0.9997 and a correlation coefficient of >0.99 (Table 5, Figure 2).

### 3.4. Tablets

Like oral capsules, tablets are also a stable dosage form that is considered practical for patient use. Four studies were retrieved. Two of them focused on Namisol, a patented tablet formulation of pure THC under investigation [54,55,56,57]. The PK data for THC doses varied from 2.5 to 8 mg, producing a Cmax of 1.42–4.69 ng/mL and a Tmax of 0.66–2.07 h in plasma. See Table 3 for the specific results.

Tablet administration showed a strong correlation between the administered THC dose and the Cmax, with a Pearson’s *r* of 0.9178 and a correlation coefficient of 0.8423 (Table 5, Figure 2).

### 3.5. Baked Goods

Five studies evaluated the THC PK after brownie or cookie consumption [38,40,58,59,61]. The THC doses in these edibles ranged from 8.4 to 50.6 mg, resulting in a Cmax of 1–16.2 ng/mL and a Tmax of 0.9–2.6 h in plasma. See Table 4 for specific results.

Brownies and cookies showed a weaker correlation between the dose of THC and the Cmax compared to other formulations, resulting in a Pearson’s *r* of 0.6365 and a correlation coefficient of 0.4051 (Table 5, Figure 2).

## 4. Discussion

The purpose of this present review was to provide a general overview of the available THC PK data after oral administration. In our literature review, we found that human PK studies studying the administration of THC in oral forms were scarce, despite their increasing popularity. Most of these studies focused on pharmaceutical forms, such as capsules and tablets. Despite being recommended formulations for the therapeutic use of cannabis in some countries, there were few data on the cannabinoid PK after the ingestion of cannabis oils or decoctions. The only complete, published study comparing these two formulations in patients with medication overuse-related headaches found high variability in the cannabinoid content of these formulations and in the THC recovery after administration. Each preparation showed differences in cannabinoid and metabolite absorption. For instance, cannabis decoctions offered a higher bioavailability of CBDA, while cannabis oil provided a higher bioavailability of THC and its metabolites, 11-hydroxy-Δ-9-tetrahidrocannabinol (11-OH-THC) and 11-nor-9-carboxy-Δ-9-tetrahidrocannabinol (THC-COOH) [52]. Contrary to these results, a published pilot study that also compared oil and decoction formulations obtained higher CBDA, THC, 11-OH-THC, and THC-COOH Cmax values after the administration of decoctions [53]. However, this pilot only presented data from one subject; results from a larger number of participants would likely strengthen comparisons between these two studies.

In our review, we found three studies that compared the oral administration of THC with that of smoking/inhalation, the most common route of administration. In Ohlsson et al. [58], subjects smoked 19.0 mg of THC (ad libitum, with a mean of 13.0 mg of THC) and took an oral dose of 20 mg of THC via a chocolate cookie. Despite being administered in a similar dose, the Cmax obtained after smoking (33–118 ng/mL) was significantly higher than the oral administration (4.4–11 ng/mL). These results showed the low systemic bioavailability of oral THC, which is about a third of that from smoked THC. Similarly, in Watchel et al. [59], the same doses of synthetic or plant-derived THC were orally administered and smoked, but the Cmax obtained was six times higher after smoking than after oral administration. As expected, the oral form achieved a delayed Tmax since absorption is slower when cannabinoids are ingested. Newmeyer et al. [60] reported minimal differences between smoked and vaporized cannabis administration, observing similar delivery. However, the THC Cmax after oral administration (brownie) was significantly lower than that of other routes, in addition to having a delayed Tmax, which agrees with previous observations.

For capsules, a wider range of doses was evaluated compared to other forms. Lile et al. administered the highest dose of THC (90 mg) out of all the studies and consequently reported the highest THC Cmax mean value obtained after administration (approx. 53 ng/mL) [49].

Oral THC doses were moderately to highly correlated (Pearson’s *r* = 0.6365–0.9997 and R^2^ = 0.4051–0.9994), with peak plasma concentrations of THC found in capsules, decoctions, tablets, and baked goods formulations (Table 5, Figure 2). This correlation was stronger for capsules, decoctions, and oil compared to baked goods, which appeared to have higher variability in the peak plasma value obtained after certain THC doses. Interestingly, for oil, the correlation was not significant, thereby suggesting a more irregular absorption profile (Figure 2). Since THC is a highly lipophilic molecule, it is expected that its absorption will increase in oil-based formulations. More studies on cannabis oil PK could determine whether this lack of linear correlation, as proven in other formulations, persists due to a high variability in the THC absorption of cannabis oil.

The management of dosing is critical for the treatment of patients, as there is a balance between the desired medical effects of THC and the prevention of adverse effects. Analyzing and understanding the PK of oral THC preparations is essential for the selection of optimal formulations, given their high variability. For instance, dronabinol in a solution exhibits lower intra-individual variability and a faster onset of detectable concentrations compared to capsule formulations [24,50].

We found that most studies on oral PK are focused on synthetic forms and analogues of THC, without considering the other cannabinoids usually present in plant-derived products, thus disregarding their possible therapeutic contributions. Moreover, the presence of CBD and other cannabinoids contained in oral cannabis preparations may be involved in alterations of THC PK properties [14].

In 2017, a review examining the PK and pharmacodynamics (PD) of oral cannabinoids for the treatment of chemotherapy-induced nausea and vomiting emphasized the high variability in the PK/PD profiles of capsules [63] and how they differ from other routes, such as smoking and intravenous delivery. The authors remarked on the efficacy of oral cannabinoids in the management of nausea and vomiting, which is similar, or even superior, to conventional antiemetic drugs. Interestingly, participants showed a preference for cannabis-based medicines over conventional medicines in trials where the two options were compared.

Similarly, a systematic review was recently conducted on CBD PK in humans, regardless of the administration route. Contrary to THC, CBD PK has been more thoroughly studied after oral and oromucosal administration (e.g., oral capsules and oromucosal sprays) than other routes of administration, such as smoking or vaporization. The most commonly studied form of administration was oromucosal spray, which contained CBD in combination with THC. Indeed, the administration of CBD alone in cannabinoid PK studies appeared less frequently than THC alone. Thus, like THC, the authors concluded that the limited available data presented some discrepancies in the PK of CBD [64].

The main limitation of the present systematic review is its use of only the PubMed database, the inclusion of only publications in English, and the exclusion of studies on the oromucosal sprays nabilone and those that administer only CBD.

## 5. Conclusions

In conclusion, oral THC has a highly variable PK profile, which differs between formulations, with seemingly higher variability in baked goods and oil forms. Considering the rapidly changing landscape of medical cannabis laws, there is an evident need for solid PK data after oral administration, especially in dosage forms other than capsules. Particularly, there is a lack of PK data on decoctions (tea) and oils, which are recommended methods of ingestion for medical use. Insufficient studies may lead to future failures of cannabis as a therapeutic compound if its therapeutic window is not defined.

The present review collects all published data on the oral administration of THC and cannabis in humans. Our results show high variability between oral formulations but a positive dose–concentration relationship for THC in most preparations.

Further investigations are required to provide more data on cannabinoid PK in the oral administration of THC, as well as other cannabinoids, to increase the accuracy when defining a therapeutic dosage for every patient.

## Figures and Tables

**Table 1:** Summary of studies included in this systematic review reporting the pharmacokinetic parameters of Δ-9-tetrahydrocannabinol (THC) following capsule administration.

References	Study Design	Participants	Route of Administration	Formulation	Dose	Cmax (ng/mL) Mean ± SD, Median (Range)	Tmax (h)	Pharmacological Effects
Wall et al., 1983 [37]	OL, NP	6 M, 6 F	Oral	Capsules	20 mg THC (men)	Men	Men	Effects were not assessed.
						THC: 14 ± 9.7 ^b^	THC: 2.5	
						11-OH-THC: 6.6 ± 3.4 ^b^	11-OH-THC: 2.0	
					15 mg THC (women)	Women	Women	
						THC: 9.4 ± 4.5 ^b^	THC: 1.75	
						11-OH-THC: 5.9 ± 2.8 ^b^	11-OH-THC: 1.75	
			Intravenous (infusion pump)	Human serum albumin	4 mg THC (men)	Men	Men	
						THC: 71 ± 34 ^b^	THC: 0.42	
						11-OH-THC: 3.7 ± 2.3 ^b^	11-OH-THC: 0.5	
					2.2 mg THC (women)	Women	Women	
						THC: 85 ± 26 ^b^	THC: 0.17	
						11-OH-THC: 3.8 ± 2.8 ^b^	11-OH-THC: 0.33	
Haney et al., 2003 [38]	R, DB, P, C	7 M Cannabis smokers	Oral	Capsules (Marinol^®^)	30 mg THC29, Placebo naltrexone	THC: 29.9 ± 9.5 ^b^	THC: 4 ^b^	*Subjective effects* Increase in ratings of Good Drug Effect, High, and Stimulated. *Vital effects* Decreased HR. Worsened psychomotor performance.
						THC-COOH: 121.9 ± 43.5 ^b^	THC-COOH: 2 ^b^	
			Oral	Capsules (Marinol^®^)	30 mg THC 50 mg naltrexone	THC: 21.2 ± 8.6 ^b^	THC: 2 ^b^	
						THC-COOH: 139.0 ± 36.2 ^b^	THC-COOH: 3 ^b^	
Naef et al., 2003 [39]	R, DB, P, C	6 M, 6 F Cannabis naïve	Oral	Capsules (Marinol^®^)	20 mg THC	THC: 7.2 ± 6.9 ^b, e^	THC: 1-2 ^b, e^	*Subjective effects* Psychotropic and somatic side-effects were common but usually mild. *Paint tests* No significant reduction in pain.
						11-OH-THC: 19.7 ± 6.9 ^b, e^	11-OH-THC: 2 ^b, e^	
						11-COOH-THC: 241.4 ± 73. ^b, e^	11-COOH-THC: 2–4 ^b, e^	
			Oral	Capsules	20 mg THC 30 mg morphine HCl	THC: 6.7 ± 7.3 ^b, e^	-	
						11-OH-THC: 7.9 ± 8.3 ^b, e^	-	
						11-COOH-THC: 134.7 ± 65.12 ^b, e^	-	
			Oral	Capsules (placebo)	30 mg morphine HCl			
			Oral	Capsules (placebo)				
Guy et al., 2004 [40]	R, OL, C followed by an NR oral dose	6 M, 6 F Previous experience of cannabis use	Oral	Capsules	10 mg THC 10 mg CBD	THC: 6.35 ± 3.12 (3.04–4.55)	THC: 1.05 ± 0.65 (0.5–2.75)	Effects were not assessed.
						CBD: 2.47 ± 2.23 (0.47–7.55)	CBD: 1.27 ± 0.84 (0.5–3)	
						11-OH-THC: 7.87 ± 2.96 (4.79–13.64)	11-OH-THC: 1.36 ± 0.63 (0.75–3)	
			Sublingual	Liquid spray (Sativex^®^)	10 mg THC 10 mg CBD	THC: 5.54 ± 3.35 (1.14–12.13)	THC: 1.63 ± 0.59 (1–3)	
						CBD: 2.50 ± 1.83 (0.27–6.55)	CBD: 1.63 ± 0.68 (0.75–3)	
						11-OH-THC: 6.24 ± 2.74 (2.67–10.77)	11-OH-THC: 1.58 ± 0.44 (1–2.75)	
			Buccal	Liquid spray (Sativex^®^)	10 mg THC 10 mg CBD	THC: 6.14 ± 5.37 (0.88–19.78)	THC: 2.40 ± 1.08 (1–4.5)	
						CBD: 3.02 ± 3.15 (0.29–9.91)	CBD: 2.78 ± 1.31 (1–4.5)	
						11-OH-THC: 6.13 ± 2.88 (1.83–11.25)	11-OH-THC: 2.40 ± 1.17 (1–4.5)	
			Oro-pharyngeal	Liquid spray (Sativex^®^)	10 mg THC 10 mg CBD	THC: 6.11 ± 4.00 (1.94–15.68)	THC: 2.23 ± 1.52 (0.75–5)	
						CBD: 2.61 ± 1.91 (0.41–6.36)	CBD: 2.04 ± 1.13 (0.75–5)	
						11-OH-THC: 6.45 ± 2.91 (2.95–13.49)	11-OH-THC: 2.40 ± 1.22 (1.25–5)	
Menetrey et al., 2005 [41]			See also Table 2 for results on capsules and decoction administration.					
Nadulski et al., 2005 [42]	DB, P, C	24	Oral	Capsules	10 mg THC 5.4 mg CBD	THC: 4.05 (1.18–10.27)	THC: 0.93 (0.55–2.08)	Effects were not assessed.
						CBD: 0.95 (0.30–2.57)	CBD: 0.99 (0.5–2)	
						11-OH-THC: 4.88 (1.83–12.34)	11-OH-THC: 1.67 (0.62–2.17)	
						THC-COOH:35.46 (19.2–70.6)	THC-COOH: 1.92 (1.08–3.83)	
			Oral	Capsules	10 mg THC	THC: 3.20 (0.67–7.99)	THC: 1.06 (0.5–3.05)	
						11-OH-THC: 4.48 (1.12–11.14)	11-OH-THC: 1.5 (0.5–3.17)	
						THC-COOH: 32.9 (12.03–57.63)	THC-COOH: 2.07 (0.62–3.92)	
			Oral	Capsules	Placebo			
Goodwin et al., 2006 [43]			See also Table 2 for results on capsules and oil administration.					
Schwilke et al., 2009 [44]	NR, OL, NP, MD	6 M Daily smokers (positive in cannabinoids, smoked within the previous 24 h)	Oral	Capsules (Marinol^®^)	Escalating total daily doses (40-120 mg) for 7 days First dose 20 mg THC	After 1st dose (single dose):	After 1st dose:	Effects were not assessed.
						THC: 12.4 ± 3.4	THC: 2.8 (0.33)	
						11-OH-THC: 8.2 ± 2.0	11-OH-THC: 2.5 (0.18)	
						THC-COOH: 75.8 ± 9.4	THC-COOH: 3.3 (0.56)	
Karschner et al., 2011 [45]	R, DB, P, DD	6 M, 3 F Cannabis smokers	Oral	Capsules (dronabinol)	5 mg THC	THC: 4.7 ± 0.9, 4.6 (1.4–10.4)	THC: 3.2 ± 0.3, 3.1 (1.5–4.5)	Effects were not assessed.
						11-OH-THC: 3.0 ± 0.4, 2.6 (1.8–5.9)	11-OH-THC: 3.3 ± 0.4, 3.3 (1.5–5.6)	
						THC-COOH: 69.3 ± 17.6, 57.1 (15.9–179.7)	THC-COOH: 4.4 ± 0.5, 4.3 (2.7–7.5)	
			Oral	Capsules (dronabinol)	15 mg THC	THC: 14.3 ± 2.7, 11.2 (3.3–28.5)	THC: 3.4 ± 0.5, 3.4 (1.2–5.5)	
						11-OH-THC: 11.1 ± 2.0, 9.3 (3.6–19.5)	11-OH-THC: 3.4 ± 0.4, 3.6 (1.0–5.5)	
						THC-COOH: 133.6 ± 36.3, 102.1 (44.5–409.0)	THC-COOH: 4.9 ± 0.5, 5.5 (2.4–7.5)	
			Sublingual	Spray (Sativex^®^)	5.4 mg THC 5.0 mg CBD	THC: 5.1 ± 1.0, 5.1 (1.2–9.6)	THC: 3.3 ± 0.3, 3.5 (1.2–4.5)	
						CBD: 1.6 ± 0.4, 1.2 (0.6–3.9)	CBD: 3.7 ± 0.5, 3.6 (1.0–5.5)	
						11-OH-THC: 4.2 ± 0.7, 3.7 (2.1– 7.5)	11-OH-THC: 3.6 ± 0.6, 3.3 (1.0–7.5)	
						THC-COOH: 108.0 ± 30.5, 79.8 (19.1–281.6)	THC-COOH: 4.4 ± 0.7, 4.5 (1.2–7.5)	
			Sublingual	Spray (Sativex^®^)	16.2 mg THC 15.0 mg CBD	THC: 15.3 ± 3.4, 14.5 (3.2–38.2)	THC: 4.0 ± 0.5, 4.5 (1.2–5.6)	
						CBD: 6.7 ± 2.0, 3.7 (2.0–20.5)	CBD: 4.0 ± 0.5, 4.5 (1.2–5.6)	
						11-OH-THC: 8.4 ± 1.2, 7.6 (3.8–13.7)	11-OH-THC: 3.9 ± 0.5, 3.7 (1.2–5.6)	
						THC-COOH: 126.6 ± 25.9, 92.4 (55.9–304.1)	THC-COOH: 4.8 ± 0.3, 5.0 (2.6–5.6)	
Karschner et al., 2012 [46]	NR, OL, NP, MD	10 M Daily smokers (positive cannabinoids, smoked within the previous 24 h)	Oral	Capsules (Marinol^®^)	Escalating total daily doses (40-120 mg) for 7 days Each dose of 20 mg THC	After 1st dose (single dose):	After 1st dose:	Effects were not assessed.
						THC: 8.7 ± 4.8, 6.4 (4.1–17.5)	THC: 3.0 ± 0.9, 3.0 (2.0–4.0)	
						11-OH-THC: 4.0 ± 2.1, 3.4 (1.8–7.8)	11-OH-THC: 2.8 ± 0.9, 3.0 (2.0–5.0)	
						THC-COOH: 38.4 ± 15.9, 36.6 (19.7–68.7)	THC-COOH: 3.1 ± 1.0, 3.0 (2.0–5.0)	
Martin-santos et al., 2012 [47]	R, DB, P, C	16 M Previous experience of cannabis use (less than 15 times in their lifetime)	Oral	Capsules	10 mg THC	THC: 0.67 ± 0.66 ^b^ THC-COOH: ≈ 5.6 ^b, d^ 11-OH-THC: ≈ 0.73 ^b, d^	THC: 2 h ^b^	*Subjective effects* Significant changes in PANSS, anxiety (STAI-S), dysphoria (ARCI), sedation (VAMS, ARCI), and the level of subjective intoxication (ASI, ARCI). *Vital effects* Significant increase in HR No significant differences in SBP and DBP.
			Oral	Capsules	600 mg CBD			
			Oral	Capsules (placebo)				
Eichler et al., 2012 [48]	R, DB, C	9 M Non smokers	Oral	Capsules (Marinol^®^)	20 mg THC	THC: 1.03 ± 1.65, 0.48 ^e, g^	THC: 1.06 ± 0.19, 1.0 ^e^	*Subjective effects* Mild psychotropic effects, with no significant differences between treatments.
						CBD: 0.00 ± 0.00, 0.0 ^e, g^	CBD: NA	
						11-OH-THC: 0.99 ± 0.63, 0.84 ^e, g^	11-OH-THC: 1.67 ± 0.51, 2.0 ^e^	
						THC-COOH: 7.13 ± 5.64, 7.61 ^e, g^	THC-COOH: 1.78 ± 0.96, 2.0 ^e^	
						CBN: 0.64 ± 0.72, 0.37 ^e, g^	CBN: 1.06 ± 0.57, 1.0 ^e^	
			Oral	Capsules (extract from heated Herba Cannabis)	17.6 mg THC 27.8 mg CBD	THC: 0.42 ± 0.39, 0.25 ^e, g^	THC: 0.78 ± 0.27, 1.0 ^e^	
						CBD: 0.30 ± 0.21, 0.27 ^e, g^	CBD: 0.83 ± 0.51, 0.5 ^e^	
						11-OH-THC: 0.73 ± 0.69, 0.50 ^e, g^	11-OH-THC: 1.44 ± 0.69, 2.0 ^e^	
						THC-COOH: 5.81 ± 7.59, 3.46 ^e, g^	THC-COOH: 2.89 ± 1.05, 2.0 ^e^	
						CBN: 0.60 ± 0.36, 0.56 ^e, g^	CBN: 0.94 ± 0.45, 1.0 ^e^	
			Oral	Capsules (extract from unheated Herba Cannabis)	10.4 mg THC 14.8 mg CBD	THC: 1.02 ± 0.78, 0.71 ^e, g^	THC: 1.17 ± 0.66, 1.0 ^e^	
						CBD: 1.24 ± 0.87, 0.96 ^e, g^	CBD: 1.17 ± 1.17, 1.0 ^e^	
						11-OH-THC: 0.57 ± 0.42, 0.50 ^e, g^	11-OH-THC: 1.00 ± 0.42, 1.0 ^e^	
						THC-COOH: 1.94 ± 1.11, 2.28 ^e, g^	THC-COOH: 2.11 ± 0.78, 2.0 ^e^	
						CBN: 0.54 ± 0.30, 0.58 ^e, g^	CBN: 1.00 ± 0.42, 1.0 ^e^	
Lile JA et al., 2013 [49]	B, P, C	4 M, 3 F Only 5 completed all doses Regular cannabis use	Oral	Capsules (Marinol^®^)	15 mg THC	THC: ≈5 ^d^	THC: 3 ^d^	*Vital effects* Increase in HR. SBP decreased after 30 mg dose but increased after 75 and 90 mg doses. No changes in DBP. Decrease in finger temperature. *Psychomotor performance* Worsened psychomotor performance.
						11-OH-THC: ≈2-3 ^d^	11-OH-THC: 3 ^d^	
			Oral	Capsules (Marinol^®^)	30 mg THC	THC: ≈10 d	THC: 3 ^d^	
						11-OH-THC: ≈5 ^d^	11-OH-THC: 3 ^d^	
			Oral	Capsules (Marinol^®^)	45 mg THC	THC: ≈17–18 ^d^	THC: 2.5 ^d^	
						11-OH-THC: ≈8–9 ^d^	11-OH-THC: 2 ^d^	
			Oral	Capsules (Marinol^®^)	60 mg THC	THC: ≈45 d	THC: 3.5 ^d^	
						11-OH-THC: ≈11 ^d^	11-OH-THC: 3 ^d^	
			Oral	Capsules (Marinol^®^)	75 mg THC	THC: ≈42–43 d	THC: 4 ^d^	
						11-OH-THC: ≈12–13 ^d^	11-OH-THC: 4 ^d^	
			Oral	Capsules (Marinol^®^)	90 mg THC	THC: ≈53 d	THC: 4 d	
						11-OH-THC: ≈20 ^d^	11-OH-THC: 4 ^d^	
			Oral	Capsules (placebo)				
Parikh et al., 2016 [50]	R, OL, C	51 MF No cannabis use in the previous 90 days	Oral	Oral solution (Dronabinol)	4.25 mg THC	THC Replicate 1: 1.81 ± 1.26	THC Replicate 1: 1.50 (0.50–4.00)	Effects were not assessed.
						THC Replicate 2: 2.08 ± 1.30	THC Replicate 2: 1.00 (0.50–3.02)	
						11-OH-THC Replicate 1: 2.53 ± 1.38	11-OH-THC Replicate 1: 1.50 (0.75–4.00)	
						11-OH-THC Replicate 2: 3.01 ± 1.56	11-OH-THC Replicate 2: 1.50 (0.50–3.02)	
			Oral	Capsules (Dronabinol)	5 mg THC	THC Replicate 1: 2.20 ± 1.51 THC Replicate 2: 2.61 ± 1.69 11-OH-THC Replicate: 3.28 ± 1.78 11-11-OH-THC Replicate 2: 3.98 ± 2.51	THC Replicate 1: 1.00 (0.50–6.00) THC Replicate 2: 1.50 (0.50–6.00) 11-OH-THC Replicate 1: 1.60 (0.75–6.00) 11-OH-THC Replicate 2: 1.50 (0.50–6.00)	
Cherniakov et al., 2017 [51]	OL, C	9 M Not exposed within the previous 4 weeks	Sublingual	Spray (Sativex^®^)	10.8 mg THC 10.0 mg CBD	THC: 1.8 ± 0.2	THC: 2 (1–4)	Effects were not assessed.
						CBD: 0.5 ± 0.1	CBD: 3 (1–5)	
			Oral	THC-CBD-piperine-PNL capsule	10.8 mg THC 10.0 mg CBD	THC: 5.4 ± 0.01	THC: 1 (1–1.5)	
						CBD: 2.1 ± 0.4	CBD: 1 (0.5–1.5)	

Abbreviations: Cmax, maximum concentration after administration; SD, standard deviations; R, randomized; NR, not-randomized; OL, open label; DB, double-blind; B, blind; P, placebo-controlled; NP, not placebo-controlled; C, crossover; DD, double-dummy; MD, multiple dose; M, male; F, female; THC, Δ-9 tetrahydrocannabinol; THCA, Δ-9-tetrahydrocannabinolic acid A; CBD, cannabidiol; CBDA, cannabidiolic acid; 11-OH-THC, 11-hydroxy-THC; THC-COOH, 11-nor-9-carboxy-THC; THC-COOH-gluc, THC–COOH–glucuronide; THCV-COOH, 11-nor-9-carboxy-tetrahydrocannabivarin; BP, blood pressure; SBP, systolic blood pressure; DBP, diastolic blood pressure; HR, heart rate; ARCI, Addiction Research Center Inventory; VAS, visual analogue scales; VAMS, visual analogue mood scale; PANSS, Positive And Negative Symptom Scale; STAI, State-Trait Anxiety Inventory; ASI, Addiction Severity Index. ^a^ Range corresponds to the range of Cmax. ^b^ The maximum value of the time-course of plasma THC. ^c^, the median instead of mean. ^d^, data deduced from a figure. ^e^, the original values for values presented as the standard error or coefficient of variation of the mean have been transformed in the table to standard deviation. ^f^, the mean values have been calculated from the values reported in the article. ^g^, values converted from pmol/mL to ng/mL.

**Table 2:** Summary of studies included in this systematic review reporting the pharmacokinetic parameters for THC following oil or decoction administration.

References	Study Design	Participants	Route of Administration	Formulation	Dose	Cmax (ng/mL) Mean ± SD, Median (Range)	Tmax (h)	Pharmacological Effects
Menetrey et al., 2005 [41]	R, DB, P, C	8 M Occasional cannabis smokers	Oral	Milk decoction	16.5 mg THC	THC: 3.8 (1.5–8.3) b	THC: 1 b	*Subjective effects* Prototypical effects of THC with a strong feeling of highness. *Vital effects* Slight to moderate conjunctival reddening. Slight to moderate tachycardia Increase of HR after decoction.
						11-OH-THC: 4.7 (2.9–7.0) b	11-OH-THC: 1 b	
						THC-COOH: 27.8 (14.1–42.4) b	THC-COOH: 4 b	
			Oral	Milk decoction	45.7 mg THC	THC: 8.4 (3.9–13.1) b	THC: 1 b	
						11-OH-THC: 12.8 (3.4–24.7) b	11-OH-THC: 2.5 b	
						THC-COOH: 66.2 (29.0–99) b	THC-COOH: 2.5 b	
			Oral	Capsules (Marinol^®^)	20 mg THC	THC: 2.8 (nd–5.6) b	THC: 1 b	
						11-OH-THC: 3.9 (1.4–8.5) b	11-OH-THC: 4 b	
						THC-COOH: 27.8 (5.4–55.4) b	THC-COOH: 5.5 b	
			Oral	Decoction (placebo)	0.8 mg THC			
			Oral	Capsules (placebo)				
Goodwin et al., 2006 [43]	R, DB, P, DD, MD (5 days)	6 Previous experience of cannabis use	Oral	Hemp oil	0.39 mg THC/day (tablespoon)	THC: 0 f (0.0–0.0)	THC: 0.0 f (0.0–0.0)	*Subjective effects* Mild prototypical effects of THC. *Vital effects* No difference in BP, HR, and respiratory rate.
						11-OH-THC: 0 f (0.0–0.0)	11-OH-THC: 0.0 f (0.0–0.0)	
						THC-COOH: 1.1 f (0.0–3.1)	THC-COOH: 49.7 f (4.5–121)	
			Oral	Capsules (hemp oil)	0.47 mg THC/day	THC: 0.0 f (0.0–0.0)	THC: 0.0 f (0.0–0.0)	
						11-OH-THC: 0.0 f (0.0–0.0)	11-OH-THC: 0.0 f (0.0–0.0)	
						THC-COOH: 1.4 f (0.0–2.6)	THC-COOH: 65.3 f (11.0–107)	
			Oral	Capsules (dronabinol)	7.5 mg THC/day	THC: 1.5 f (0.6–3.8)	THC: 57.6 f (6.5–107)	
						11-OH-THC: 1.6 f (0.0–2.6)	11-OH-THC: 85.9 f (1.5–107)	
						THC-COOH: 19.8 f (10.6–43.0)	THC-COOH: 107 f (107–107)	
			Oral	Hemp oil	14,8 mg THC/day	THC: 2.1 f (0.7–6.1)	THC: 56.5 f (9–107)	
						11-OH-THC: 1.7 f (0.0–5.6)	11-OH-THC: 28.6 f (6.5–107)	
						THC-COOH: 12.7 f (11.0–15.2)	THC-COOH: 91.5 f (11.5–121)	
			Oral	Placebo				
Pellesi et al., 2018 [52]	OL, C	6 M, 7 F Patients with medication overuse headaches	Oral	Decoction	1.85 ± 1.6 mg THC	THC: 1.38 ± 0.75	THC: 1.28 ± 0.51	*Subjective effects* Intensity of subjective effects was similar in both formulations. Increased drowsiness after cannabis oil administration. *Vital effects* No changes in BP and HR.
					2.22 ± 0.66 mg THCA-A	THCA: 48.92 ± 26.34	THCA: 1.22 ± 0.26	
					1.93 ± 1.17 mg CBD	CBD: 4.39 ± 3.01	CBD: 0.56 ± 0.17	
					8.82 ± 2.02 mg CBDA	CBDA: 74.61 ± 25.15	CBDA: 0.83 ± 0.35	
			Oral	Oil	2.2 mg THC	THC: 3.29 ± 1.39	THC: 1.28 ± 0.36	
					2.3 mg THCA-A	THCA: 65.36 ± 20.40	THCA: 1.33 ± 0.35	
					2.4 mg CBD	CBD: 3.14 ± 2.58	CBD:1 ± 0.25	
					4.4 mg CBDA	CBDA: 55.03 ± 29.45	CBDA: 1.06 ± 0.3	
Pichini et al., 2020 [53]	NR, OL, NP Pilot	1 M	Oral	Decoction	0.36 mg THC 1.6 mg THCA-A 0.42 mg CBD 4 mg CBDA	Blood	Blood	Effects were not reported.
						THC: 1.0	THC: 2.0	
						THCA-A: 72.4	THCA-A: 2.0	
						CBD: 1.5	CBD: 3.0	
						CBDA: 94.3	CBDA: 0.5	
						11-OH-THC: 1.2	11-OH-THC: 2.0	
						THC-COOH: 17.1	THC-COOH: 3.0	
						THC-COOH-GLUC: 40.2	THC-COOH-GLUC: 4.0	
						Oral fluid	Oral fluid	
						THC: 0.2	THC: 0.5	
						THCA-A: 5.1	THCA-A: 0.5	
						CBD: 0.8	CBD: 0.5	
						CBDA: 145.2	CBDA: 0.5	
			Oral	Oil	0.95 mg THC 1.5 mg THCA-A 0.86 mg CBD 2.8 mg CBDA	Blood	Blood	
						THC: 0.5	THC: 2.0	
						THCA: 40.3	THCA-A: 2.0	
						CBD: 0.3	CBD: 2.0	
						CBDA: 32.4	CBDA: 1.5	
						11-OH-THC: 0.7	11-OH-THC: 2.0	
						THC-COOH: 4.3	THC-COOH: 2.0	
						THC-COOH-GLUC: 7.7	THC-COOH-GLUC: 3.0	
						Oral fluid	Oral fluid	
						THC: 0.2	THC: 2	
						THCA: 1.0	THCA-A: 2	
						CBD: 0.6	CBD: 2	
						CBDA: 14.3	CBDA: 1	

For abbreviations see Table 1.

**Table 3:** Summary of studies included in this systematic review reporting the pharmacokinetic parameters of THC following tablet administration.

References	Study Design	Participants	Route of Administration	Formulation	Dose	Cmax (ng/mL) Mean ± SD, Median (Range)	Tmax (h)	Pharmacological Effects
Timpone et al., 1997 [54]	R, OL	7 M/F 4 M/F 9 M/F Patients with HIV wasting syndrome	Oral	Tablets (Marinol^®^)	2.5 mg THC	Data from all 20 patients	Data from all 20 patients	*Subjective effects* Increase in VAS for hunger. No differences in VAS for mood and nausea.
			Oral	Tablets (Marinol^®^)	2.5 mg THC 750 mg megestrol	*THC: 2.01 c (0.58–12.48)*	*THC: 2.07 b (0.66–8.26)*	
			Oral	Tablets (Marinol^®^)	2.5 mg THC 250 mg megestrol	*11-OH-THC: 4.61 c (0.52–37.5)*	*11-OH-THC: 2.07 b (0.49–8.00)*	
Klumpers et al., 2011 [55]	R, DB, DD, P, C	4 M, 5 F (in panel 1, 13 subjects) Previous experience of cannabis use (maximum 1 use per week)	Sublingual	Tablets (Namisol^®^)	5.0 mg THC	2.30 ± 1.01 ^e^	1.24 ± 0.65 ^e^	*Subjective effects* Highest oral doses increased body sway and VAS for calmness, external perception, and feeling high and decreased VAS for alertness. *Vital effects* No significant differences in PD parameters between oral and sublingual administration. Significant increase in HR.
			Oral	Tablets (Namisol^®^)	5.0 mg THC	2.92 ± 1.49 ^e^	0.93 ± 0.68 ^e^	
			Oral	Tablets (Namisol^®^)	6.5 mg THC	4.43 ± 1.86 ^e^	0.66 ± 0.13 ^e^	
			Oral	Tablets (Namisol^®^)	8.0 mg THC	4.69 ± 2.91 ^e^	0.73 ± 0.19 ^e^	
			Oral	Tablets (placebo)				
Ahmed et al., 2014 [56]	R, DB, P, DD, C	6 M, 5 F	Oral	Tablets (Namisol^®^)	3 mg THC	1.42 (0.53–3.48)	0.92 (0.67–0.92)	*Subjective effects* No subjective effects (exc. 4 subjects “felt high”) *Vital effects* Mild PD effects. No changes in SBP, DBP, and HR. *Psychomotor performance* No changes in psychomotor performance.
			Oral	Tablets (Namisol^®^)	5 mg THC	3.15 (1.54–6.95)	0.92 (0.67–0.92)	
			Oral	Tablets (Namisol^®^)	6.5 mg THC	4.57 (2.11–8.65)	0.67 (0.67–0.92)	
			Oral	Tablets (placebo)				
De Vries et al., 2016 [57]	R, DB, P, C	15 M, 9 F Patients diagnosed with chronic pancreatitis No cannabis use in previous year	Oral	Tablets (Namisol^®^)	8 mg THC	THC: 4.01 ± 3.39	2.05 + 1.47	*Subjective effects* No differences in subjective effects (alertness, mood, calmness, or balance) between treatments. Anxiousness, somnolence, dry mouth, dizziness, and euphoric mood after THC administration. *Vital effects* No changes in SBP and DBP. THC induced an increase in HR compared to diazepam.
						11-OH-THC: 4.38 ± 1.50	2.26 ± 1.29	
			Oral	Tablet (active placebo)	5 mg diazepam to non-opioid group/10 mg diazepam to opioid group			

For abbreviations see Table 1.

**Table 4:** Summary of studies included in this systematic review reporting the pharmacokinetic parameters for THC following baked goods’ administration.

References	Study Design	Participants	Route of Administration	Formulation	Dose	Cmax (ng/mL) Mean ± SD, Median (Range)	Tmax (h)	Pharmacological Effects
Ohlsson et al., 1980 [58]	R, OL, NP, C	11 M Previous experience of cannabis use (from infrequent to frequent use)	Smoked	Cigarette	19 mg THC (ad libitum) (mean = 13.0 mg)	77, 33–118 ^a^		*Subjective effects* Increase in high effect. *Vital effects* Increase in HR. Conjunctival reddening
			Oral	Chocolate cookie	20 mg THC	4.4–11 ^a^	1–1.5	
			Intravenous	Normal saline ethanolic solution	5 mg THC	219, 161–316 ^a^		
Watchel et al., 2002 [59]	DB, P, C	7 M, 5 F 7 M, 6 F Previous experience of cannabis use	Oral	Cannabis (plant) brownie	8.4 mg THC	≈ 4.1 ^b, d^	3 ^b, d^	*Subjective effects* Both drugs increased VAS sedation and ARCI PCAG scale scores, and decreased the ARCI BG scale scores at higher doses. Cannabis in high doses increased VAS for sedation, drowsiness, and tiredness. THC in high doses increased ARCI A scale scores, MBG (euphoria), and LSD (dysphoria). *Vital effects* No effects on physiological or behavioral measures.
			Oral	Cannabis (plant) brownie	16.9 mg THC	≈ 6.8 ^b, d^	2.5 ^b, d^	
			Oral	THC (synthetic) brownie	8.4 mg THC	≈ 4.8 ^b, d^	2.5 ^b, d^	
			Oral	THC (synthetic) brownie	16.9 mg THC	≈ 9 ^b, d^	2.5 ^b, d^	
			Smoked	Cannabis (plant) cigarette	8.4 mg THC	≈ 36 ^b, d^	0.08 ^b, d^	
			Smoked	Cannabis (plant) cigarette	16.9 mg THC	≈ 60 ^b, d^	0.08 ^b, d^	
			Smoked	THC (synthetic) cigarette	8.4 mg THC	≈ 31 ^b, d^	0.08 ^b, d^	
			Smoked	THC (synthetic) cigarette	16.9 mg THC	≈ 56 ^b, d^	0.08 ^b, d^	
			Oral	Brownie (placebo)				
			Smoked	Cigarette (placebo)				
Newmeyer et al., 2016 [60]	R, DB, P, DD, C	9 M, 2 F frequent smokers 6 M, 3 F occasional smokers	Oral	Brownie	50.6 mg THC (ad libitum) 1.5 mg CBD 3.3 mg CBN	Frequent smokers	Frequent smokers	Effects were not described.
						THC: 15.3, 14.3 (1.4–32.4)	THC: 2.5, 2.5 (1.5–3.5)	
						11-OH-THC: 7.3, 6.2 (0.9–13.7)	11-OH-THC: 2.3, 2.5 (1.5–3.5)	
						THC-COOH: 36.4, 35.3 (4.3–99.4)	THC-COOH: 2.7, 2.5 (2.5–3.5)	
						THCV-COOH: 2.1, 2.0 (1.1–3.4)	THCV-COOH: 3.0, 3.0 (2.5–3.5)	
						THC-COOH-gluc: 53.0, 57.1 (10.3–75.7)	THCOOH-gluc: 3.4, 3.5 (1.5–5.0)	
						Occasional smokers	Occasional smokers	
						THC: 10.3, 10.1 (3.6–22.5)	THC: 2.3, 2.5 (1.5–3.5)	
						11-OH-THC: 5.5, 5.1 (2.4–11.0)	11-OH-THC: 2.4, 2.5 (1.5–3.5)	
						THC-COOH: 39.8, 37.8 (12.5–70.4)	THC-COOH: 2.9, 3.5 (1.5–3.5)	
						THCV-COOH: 1.9, 1.9 (1.1–2.7)	THCV-COOH: 2.6, 2.5 (1.5–3.5)	
						THC-COOH-gluc: 124, 124 (70.9–178)	THC-COOH-gluc: 4.7, 5.0 (3.5–5.0)	
			Smoked	Cigarette	50.6 mg THC (ad libitum) 1.5 mg CBD 3.3 mg CBN	Frequent smokers	Frequent smokers	
						THC: 151, 114 (51.6–467)	THC: 0.12, 0.13 (0.00–0.17)	
						11-OH-THC: 9.0, 6.5 (1.9–30.2)	11-OH-THC: 0.21, 0.20 (0.10–0.50)	
						THC-COOH: 23.5, 20.0 (5.7–64.9)	THC-COOH: 0.28, 0.25 (0.00–0.50)	
						THCV-COOH: 2.4, 2.4 (1.8–3.1)	THCV-COOH: 0.22, 0.23 (0.17–0.25)	
						THC-COOH-gluc: 25.8, 14.1 (5.0–70.7)	THC-COOH-gluc: 1.1, 0.5 (0.0–3.5)	
						Occasional smokers	Occasional smokers	
						THC: 51.6, 44.4 (1.3–174)	THC: 0.11, 0.10 (0.07–0.17)	
						11-OH-THC: 2.8, 1.9 (0.5–8.7)	11-OH-THC: 0.22, 0.19 (0.10–0.50)	
						THC-COOH: 8.4, 7.4 (0.7–17.5)	THC-COOH: 0.31, 0.25 (0.10–0.50)	
						THCV-COOH: -	THCVCOOH: -	
						THC-COOH-gluc: 19.4, 21.4 (11.8–25.0)	THC-COOH-gluc: 2.1, 1.5 (1.5–3.5)	
			Inhaled	Vaporizer Volcano	50.6 mg THC (ad libitum) 1.5 mg CBD 3.3 mg CBN	*Frequent smokers*	Frequent smokers	
						THC: 84.7, 83.1 (23.5–169)	*THC: 0.09, 0.10 (0.03–0.17)*	
						11-OH-THC: 4.8, 4.2 (1.6–9.8)	*11-OH-THC: 0.19, 0.17 (0.10–0.50)*	
						THC-COOH: 13.0, 12.5 (4.1–31.3)	*THC-COOH: 0.25, 0.25 (0.13–0.50)*	
						THCV-COOH: 1.7, 1.8 (1.2–2.1)	*THCV-COOH: 0.52, 0.21 (0.17–1.5)*	
						THC-COOH-gluc: 10.9, 10.6 (0.8–23.8)	*THC-COOH-gluc: 1.8, 1.5 (0.03–3.5)*	
						*Occasional smokers*	*Occasional smokers*	
						THC: 47.8, 34.8 (5.2–137)	*THC: 0.11, 0.10 (0.03–0.17)*	
						11-OH-THC: 2.0, 1.6 (0.7–3.5)	*11-OH-THC: 0.15, 0.15 (0.10–0.20)*	
						THC-COOH: 7.2, 5.3 (1.4–15.9)	*THC-COOH: 0.33, 0.25 (0.20–0.50)*	
						THCV-COOH: -	*THCV-COOH: -*	
						THC-COOH-gluc: 15.1, 16.1 (5.3–23.7)	*THC-COOH-gluc: 1.9, 2.5 (0.5–2.5)*	
			Oral	Brownie (placebo)				
			Smoked	Cigarette (placebo)				
			Inhaled	Vaporizer (placebo)				
Newmeyer et al., 2017 [61]	Optional dosing session under the same clinical protocol followed in Newmeyer et al., 2016	9 M frequent smokers 5 M, 2 F occasional smokers	Oral	Brownie	50.6 mg THC 1.5 mg CBD 3.3 mg CBN (ad libitum)	*Frequent smokers*	*Frequent smokers*	Effects were not assessed.
						Blood	Blood	
						THC: 16.2, 12.8 (5.3–34.6)	THC: 2.5, 3.5 (1.0–3.5)	
						11-OH-THC: 58.4, 50.0 (27.8–152)	11-OH-THC: 2.8, 3.5 (1.0–3.5)	
						THC-COOH: 58.4, 50.0 (27.8–152)	THC-COOH: 3.3, 3.5 (1.5–3.5)	
						THCV-COOH: 1.9, 1.6 (1.1–3.9)	THCV-COOH: 3.1, 3.5 (1.5–3.5)	
						THC-COOH-gluc: 68.5, 61.2 (50.6–110)	THC-COOH-gluc: 4.8, 5.0 (3.5–8.0)	
						Oral fluid	Oral fluid	
						THC: 573, 464 (39.3–2111)	THC: 0.33	
						11-OH-THC: 0.6, 0.7 (0.2–1.2)	11-OH-THC: 0.40, 0.33 (0.33–1.0)	
						THC-COOH: 285, 186 (123–849)	THC-COOH: 12, 5 (3.5–48)	
						THCV-COOH: 7.4, 6.8 (1.3–19.4)	THCV-COOH: 0.33	
						*Occasional smokers*	*Occasional smokers*	
						Blood	Blood	
						THC: 8.2, 8.6 (3.2–14.3)	THC: 2.2, 1.5 (1.0–5.0)	
						11-OH-THC: 5.6, 5.2 (4.1–8.6)	11-OH-THC: 2.6, 3.5 (1.5–3.5)	
						THC-COOH: 39.7, 38.2 (26.5–61.2)	THC-COOH: 3.2, 3.5 (1.5–3.5)	
						THCV-COOH: 1.6, 1.6 (1.1–2.1)	THCVCOOH: 2.3, 1.5 (1.0–3.5)	
						THC-COOH-gluc: 86.2, 73.5 (43.1–183)	THCOOH-gluc: 4.6, 5.0 (3.5–6.0)	
						Oral fluid	Oral fluid	
						THC: 362, 392 (115–696)	THC: 0.33	
						11-OH-THC: 0.4, 0.4 (0.3–0.6)	11-OH-THC: 0.60, 0.33 (0.33–1.5)	
						THC-COOH: 315, 191 (27.9–1263)	THC-COOH: 10, 10 (0.33–20)	
						THCV-COOH: 5.4, 4.7 (1.6–10.6)	THCV-COOH: 0.33	
Vandrey et al., 2017 [62]	R, DB, NP	9 M, 9 F Previous experience of cannabis use but not exposed within the previous 3 months	Oral	Brownie	10 mg THC	Blood	Blood	*Subjective effects* Significant subjective and cognitive drug effects at the 25 and 50 mg doses. *Vital effects* Significant PD effects. *Psychomotor performance* Significant effects on psychomotor performance at the 25 and 50 mg doses.
						THC: 1.0 (0–3)	THC: 0.9 (0–2)	
						11-OH-THC: 1.0 (0–2)	11-OH-THC: 1.3 (0–3)	
						THC-COOH: 7.2 (5–14)	THC-COOH: 3.2 (2–4)	
						Oral fluid	Oral fluid	
						THC: 191.5 (47–412)	THC: 0.2 (0.2–0.5)	
						THC-COOH: 0.051 (0–0.231)	THC-COOH: 1.0 (0–3)	
			Oral	Brownie	25 mg THC	Blood	Blood	
						THC: 3.5 (3.0–4)	THC: 2.6 (1.0–4)	
						11-OH-THC: 3.3 (2–5)	11-OH-THC: 3.0 (1.5–4)	
						THC-COOH: 21.3 (12–39)	THC-COOH: 3.3 (1.5–6)	
						Oral fluid	Oral fluid	
						THC: 477.5 (70–1128)	THC: 0.2 (0.2–0.5)	
						THC-COOH: 0.140 (0.023–0.251)	THC-COOH: 9.8 (3–30)	
			Oral	Brownie	50 mg THC	Blood	Blood	
						THC: 3.3 (1.0–5)	THC: 2.3 (1.0–6)	
						11-OH-THC: 3.2 (2–4)	11-OH-THC: 1.8 (1–3)	
						THC-COOH: 29.3 (16–44)	THC-COOH: 3.3 (1.5–6)	
						Oral fluid	Oral fluid	
						THC: 597.5 (350–1010)	THC: 0.2 (0.2–0.5)	
						THC-COOH: 0.314 (0–0.822)	THC-COOH: 17.4 (0–54)	

For abbreviations, see Table 1.

**Table 5:** Correlation between doses of THC and the maximum plasma concentration (Cmax) values in each formulation group.

	Capsules	Decoction	Oil	Tablets	Baked Goods
Pearson’s *r*	0.9271	0.9997	0.3806	0.9178	0.6365
95% confidence interval	0.8492 to 0.9656	0.9851 to 1.000	−0.9154 to 0.9824	0.6032 to 0.9853	0.09838 to 0.8866
*p* value (two-tailed)	<0.0001	0,0003	0.6194	0.0013	0.0261
R^2^	0.8596	0.9994	0.1448	0.8423	0.4051
